# Patient costs of diabetes mellitus care in public health care facilities in Kenya

**DOI:** 10.1002/hpm.2905

**Published:** 2019-10-17

**Authors:** Robinson Oyando, Martin Njoroge, Peter Nguhiu, Antipa Sigilai, Fredrick Kirui, Jane Mbui, Zipporah Bukania, Andrew Obala, Kenneth Munge, Anthony Etyang, Edwine Barasa

**Affiliations:** ^1^ Health Economics Research Unit KEMRI‐Wellcome Trust Research Programme Nairobi Kenya; ^2^ Department of Clinical Sciences Liverpool School of Tropical Medicine Liverpool UK; ^3^ Centre for Geographic Medicine Research Kenya Medical Research Institute KiIifi Kenya; ^4^ Centre for Clinical Research Kenya Medical Research Institute Nairobi Kenya; ^5^ Centre for Public Health Research Kenya Medical Research Institute Nairobi Kenya; ^6^ Medical Microbiology Moi University Eldoret Kenya; ^7^ Nuffield Department of Medicine University of Oxford Oxford UK

**Keywords:** catastrophe, diabetes mellitus, Kenya, out‐of‐pocket costs, productivity losses

## Abstract

**Objective:**

To estimate the direct and indirect costs of diabetes mellitus care at five public health facilities in Kenya.

**Methods:**

We conducted a cross‐sectional study in two counties where diabetes patients aged 18 years and above were interviewed. Data on care‐seeking costs were obtained from 163 patients seeking diabetes care at five public facilities using the cost‐of‐illness approach. Medicines and user charges were classified as direct health care costs while expenses on transport, food, and accommodation were classified as direct non–health care costs. Productivity losses due to diabetes were classified as indirect costs. We computed annual direct and indirect costs borne by these patients.

**Results:**

More than half (57.7%) of sampled patients had hypertension comorbidity. Overall, the mean annual direct patient cost was KES 53 907 (95% CI, 43 625.4‐64 188.6) (US$ 528.5 [95% CI, 427.7‐629.3]). Medicines accounted for 52.4%, transport 22.6%, user charges 17.5%, and food 7.5% of total direct costs. Overall mean annual indirect cost was KES 23 174 (95% CI, 20 910‐25 438.8) (US$ 227.2 [95% CI, 205‐249.4]). Patients reporting hypertension comorbidity incurred higher costs compared with diabetes‐only patients. The incidence of catastrophic costs was 63.1% (95% CI, 55.7‐70.7) and increased to 75.4% (95% CI, 68.3‐82.1) when transport costs were included.

**Conclusion:**

There are substantial direct and indirect costs borne by diabetic patients in seeking care from public facilities in Kenya. High incidence of catastrophic costs suggests diabetes services are unaffordable to majority of diabetic patients and illustrate the urgent need to improve financial risk protection to ensure access to care.

## 1 BACKGROUND

Diabetes mellitus (DM) is a chronic, incurable, and potentially disabling disease that presents a substantial public health challenge worldwide.^1^ Evidence suggests that the unprecedented increase in DM burden has major clinical, economic, and social implications particularly in low‐ and middle‐income countries (LMICs).^2^, ^3^, ^4^, ^5^, ^6^, ^7^ In addition to reducing well‐being, the chronic nature and complications associated with DM may lead to substantial costs of medical care and productivity losses to patients and their households. Unfortunately, progress in attainment of Sustainable Development Goal 3.4 that aims to reduce premature mortality from noncommunicable diseases (NCDs) by one‐third by 2030 continues to be constrained by, inter alia, weak health systems in most LMICs.^1^

The Kenyan health system is devolved, with the National Ministry of Health (MOH) having policy and regulatory roles while the 47 county health systems have service provision roles.^8^ Service delivery is pluralistic and is characterized by a mix of public, private for‐profit, and private not‐for‐profit providers.^9^, ^10^ Kenya's public health care delivery system is organized into six levels. Level 1 is composed of community health services that include all community‐based demand creation activities that are guided by the MOH community strategy. Levels 2 and 3 refer to dispensaries and health centers, respectively, which offer outpatient primary health care services. Level 4 represents subcounty hospitals that are first referral hospitals while level 5 represents county referral hospitals that provide secondary care. Level 6 represents national tertiary referral hospitals. Diabetes care is typically offered through dedicated specialized clinics located in public levels 4 to 6 hospitals.^11^, ^12^, ^13^, ^14^ In some areas, however, patients can access medication from health centres and dispensaries, but this is the exception rather than the norm. Each of these levels are expected to provide some aspects of preventive, promotive, curative, and rehabilitative services as outlined in the Kenya Essential Package for Health,^15^ which includes interventions and services targeted at DM. Private providers mimic this classification though most are stand‐alone units with weak referral mechanisms. Kenya's health system is financed by (a) tax revenues collected by the government (national and county), (b) donor funding, (c) household contributions to the National Hospital Insurance Fund (NHIF), (d) household contributions to private health insurance companies, and (d) out‐of‐pocket (OOP) payments at points of care.

The first nationally representative survey of 2015 found an age‐standardized DM prevalence of 2.4% with 3.1% of Kenya's population having impaired fasting glycemia.^12^, ^16^ The increasing prevalence and the chronic nature of DM makes it a costly disease both to Kenya's health system and the affected households as it has been shown that persons with diabetes incur up to three times higher medical costs compared with nondiabetics.^17^, ^18^, ^19^ In addition, delay in diagnosis, poor quality of care or the lack thereof, presence and severity of complications, and comorbid conditions are the most important factors related to DM care costs.^20^, ^21^

Evidence of patient costs associated with DM care is needed to assess the economic impact of DM to households, the extent to which DM patients and households are protected from financial hardship due to health care use and to design effective financial risk protection mechanisms for this group of patients.^6^, ^21^, ^22^ We therefore conducted this study to document the patient costs of DM at primary care level in Kenya.

## 2 METHODS

### 2.1 Study setting

The study was conducted from June to December 2017 in two sites in Kenya (Kilifi and Bungoma County) purposively selected to reflect a diverse set of demographic, socio‐economic, and geographical settings. Kilifi is located on the coast of Kenya, and a high burden of stroke and heart failure has been described in this area.^23^ The population in Kilifi has been well characterized by data from the health and demographic surveillance system run by the KEMRI Wellcome Trust Research Programme.^24^ The Webuye Health and Demographic Surveillance System run by Moi University is located in Bungoma County in the western region of Kenya.^25^ Multiple cardiovascular risk factors have been identified in this area.^26^ Table 1 outlines the study site characteristics.

**Table 1:** Selected study site indicators in 2017

County	Estimated Population	No. of Public Hospitals^27^	No. of Health Centers and Dispensaries^27^	No. of OPD Patients	No. of Admissions	No. of Diabetes Cases	No. of Hypertension Cases
Bungoma	1 759 499^28^	9	125	1 215 525	70 665	3038	15 908
Kilifi	1 466 856^29^	5	123	1 243 315	28 746	4663	26 458

(all data except where otherwise indicated^30^).

Six public health care facilities were purposively selected in consultation with county health officials in respective counties to generate a sample of facilities with different workloads, plus the location of the clinics relative to the communities served. However, due to the 150‐day nation‐wide nurses' strike at the time of data collection,^27^ data were collected from five facilities unlike the anticipated six facilities in the two counties. In Kilifi, a public hospital and a health center that provided DM treatment were selected, while in Bungoma, three public hospitals were sampled. For this descriptive analysis, data from all the facilities were pooled.

### 2.2 Sample size and sampling

The target enrolment was 282 patients for a sample size sufficient to obtain an estimate of DM patient costs based on the formulae by Kirkwood^28^:

$$N={\left(Z_{\alpha /2}+Z_{\beta }\right)}^{2}*\left(P\;\left(1–P\right)\right)/e^{2})$$

where *Z*_α/2_ is the critical value of the normal distribution at *α*/2 (for a confidence level of 95%, *α* is .05 and the critical value is 1.96), *Z*_*β*_ is the critical value of the normal distribution at *β* (for a power of 80%, *β* is 0.2 and the critical value is 0.84), *P* is the expected true proportion of DM in the population in Kenya of 10% (0.10), and *e* is the desired standard size of standard error around the estimated proportion of 5% (±0.05).

Every DM patient receiving treatment and available at participating facilities during data collection was approached to participate in the study. Patients were eligible if they self‐reported DM diagnosis, had received treatment for a minimum of 6 months after diagnosis and were more than 18 years of age. Consenting patients were selected based on meeting the eligibility criteria and the order of arrival at the clinic. Respondents were asked to report on their health service use, associated costs, income, and coping mechanisms if they undertook any of the following to meet DM care costs: borrowing (having taken a loan), selling household items or assets (eg, livestock), and use of savings.

### 2.3 Measuring patient costs

The cost‐of‐illness approach was used to document patient costs.^29^ Interviews were conducted using a structured questionnaire. Three trained interviewers collected the data in the two study sites following a pilot from a facility in Kilifi that was not selected in the main study. Interviews were conducted primarily in Kiswahili, with local languages (Kigiriama and Kibukusu in Kilifi and Bungoma, respectively) used to clarify questions where necessary. Respondents were asked about costs incurred for different care‐seeking episodes described in Table 2.

**Table 2:** Care‐seeking episodes included in patient cost estimates

Care‐seeking Episode	Description	Recall Period
Sick visit	Cost of current care seeking (during interview), and any out‐patient visit when the patient was ill due to DM outside the scheduled clinic appointments	1 month
Inpatient visit	Cost of admission due to DM	12 months
Drug collection visit	Cost of regular medicines prescribed to the patient to manage DM	Frequency of drug collection, ie, monthly/quarterly
Laboratory/diagnostic visits	Cost of routine laboratory/diagnostic services done at a health facility	Frequency of lab/diagnostic services, ie, monthly/quarterly
Scheduled clinic check‐up visits	Costs due to regular clinic appointments	Frequency of clinic appointments, ie, monthly/quarterly

To annuitize sick visit costs, we summed up costs incurred during current care visit and any reported outpatient visit costs that occurred due to DM in the last 4 weeks prior to the study then multiplied by 13 (assuming there are 52 weeks in a year). On the other hand, to annuitize costs in other care‐seeking episodes described in Table 2, reported costs were multiplied by the frequency of visits, i.e., weekly, monthly, or quarterly for each episode. Furthermore, any inpatient admission costs in the last 1 year were also collected. Overall DM care costs for all care‐seeking episodes were calculated by summing up the annual costs in each care‐seeking episode. Costs incurred by patients in the overall sample, costs incurred by diabetics without comorbidities (hereafter called diabetes‐only patients), and costs incurred by diabetics with comorbidities (hereafter called comorbid patients), i.e., patients who reported both DM and hypertension diagnosis, were analysed and reported separately.

For each of the care‐seeking episode, two broad cost categories were estimated: direct OOP costs and indirect (productivity losses) costs for both patients and their caregivers. Direct health care costs included any charges levied for medicines and user fees, i.e., registration, consultation, and laboratory services. Direct non–health care costs included transport costs to and from a health provider and any costs associated with food or accommodation while seeking care. For this analysis, OOP costs were defined as the sum of direct health care and direct non–health care costs. The analysis was restricted to patients who reported any OOP costs for each care‐seeking episode.

Indirect costs were estimated based on the total hours lost while seeking care as well as the cost of illness‐related to lost productivity for both patients and their caregivers, assuming that these hours would have been used for productive activity in the absence of DM.^30^ Income lost due to DM illness was therefore estimated by multiplying the estimated number of lost production hours due to DM by the official minimum wage; by the official minimum wage of Kenya shillings (KES) 8568 (US$ 84/month) in the agricultural sector in 2017 (given the main economic activities in our study sites).^31^ We assumed an average workday of 8 h/day and 22 working days/month. Caregivers' lost productivity was also estimated by multiplying the total number of hours spent caring for the patient by the official minimum wage rate.

Income was estimated by asking detailed questions about income categories, including patient income, income for household members, welfare payments, and government assistance. As a measure of financial risk protection, we compared total direct costs incurred in the overall sample and by socio‐economic status, against annual household income and total direct costs excluding transportation costs and defined costs as catastrophic if they exceeded 10% of annual household income.^32^

### 2.4 Data management and analysis

Data were double entered to enhance data quality. The two data sets were compared to eliminate data entry errors. Consistency and range checks were used to ensure the completeness of data. Data were analysed using STATA 14.0 (STATA, StataCorp, Texas). Frequency counts and percentages were used to describe patient demographic and socio‐economic variables. Due to skewed nature of costs data, mean and median values were reported for all cost estimates as a measure of central tendency and 95% confidence intervals (CI) and interquartile range (IQR) were reported. Cost results are presented in KES and US Dollars (US$). Cost in KES was converted to US$ using the following exchange rate: US$ 1 = KES 102 (average exchange rate, January‐December 2017).^33^

### 2.5 Ethical approval

Ethical approval for the study was obtained from the Scientific Ethics Review Unit (SERU) of KEMRI (Ref: KEMRI/SERU/CGMR‐C/041/3270) and from the County Department of Health in both study sites. Written consent was obtained from each participant. Facility managers were informed about the study and granted permission to access clinics and patients.

## 3 RESULTS

### 3.1 Participants characteristics and health services utilization

Overall, 163 patients were interviewed: 92 (56.4%) from Bungoma and 71 (43.6%) from Kilifi. The mean age in the sample was 58.9 years, and 58.9% of the respondents were female. More than half of the patients reported hypertension comorbidity (57.7%), were not enrolled to any health insurance scheme (68.1%), and slightly under half were diagnosed with either DM or hypertension (HTN) more than 5 years ago (47.9%) (Table 3). The median travel time to a health facility was 30 minutes (IQR, 18‐60). Forty‐five patients (27.6%) reported that the facility where they sought care was not the nearest to them; of these, 48.9% (95% CI, 34.2‐63.7) reported lack of resources such as medicines and/or diagnostic equipment, 37.8% (95% CI, 24.5‐53.2) reported referral, and 13.3% (95% CI, 5.9‐27.3) reported other reasons (eg, time consuming to wait) for not visiting the nearest facility.

**Table 3:** Patient characteristics

Characteristic	Observations (n = 163)	Proportion (95% CI)
Mean age in years	163	58.9 (56.8‐61.0)
Gender		
Male	67	41.1% (33.7‐48.9)
Female	96	58.9% (51.1‐66.2)
Illness condition		
Diabetes mellitus	69	42.3% (34.9‐50.1)
Diabetes mellitus & Hypertension	94	57.7% (49.9‐65.1)
Enrolled to a health insurance scheme		
Yes	52	31.9% (25.1‐39.5)
No	111	68.1% (60.5‐74.9)
Employment status		
Formal employment	32	19.6 (14.2‐26.5)
Informal/unemployed	131	80.4 (73.5‐85.8)
Reason for not working		
Related to DM/DM & HTN comorbidity	48	29.4% (22.9‐37.0)
Not related to DM/DM & HTN comorbidity	115	70.6% (63.0‐77.1)
Highest level of education		
None	27	16.6% (11.6‐23.2)
Primary	66	40.5% (33.2‐48.3)
Secondary	47	28.8% (22.3‐36.3)
Graduate/certificate	23	14.1% (9.5‐20.4)
Where diagnosed		
Public facility	132	81.0% (74.1‐86.3)
Mission facility	7	4.3% (2.0‐8.7)
Private facility	24	14.7% (10.0‐21.1)
Illness duration		
6 mo to <1 y	16	9.8% (6.1‐15.5)
1‐5 y	69	42.3% (34.9‐50.1)
≥5 y	78	47.9% (40.2‐55.6)

### 3.2 Patient costs associated with health care use

#### 3.2.1 Sick visit costs

In the overall sample, 36% (n = 59) of patients reported a sick visit, i.e., sought care out of their scheduled clinic appointments a month before the survey. Similarly, 29% (n = 20) of diabetes‐only patients and 41% (n = 39) of comorbid patients reported a sick visit. Mean annual costs for medicines attracted the highest direct cost in the overall sample—KES 15 340.8 (95% CI, 6120‐24 561.6) (US$ 150.4 [95% CI, 60‐240.8]) mainly driven by the contribution of medicine costs for comorbid patients—KES 17 880 (95% CI, 4641.7‐31 118.3) (US$ 175.3 [95% CI, 45.5‐305.1]). However, among diabetes‐only patients, user charges attracted the highest mean annual cost—KES 12 733.5 (95% CI, 1825‐27 292) (US$ 124.8 [95% CI, 17.9‐267.6]) (Table 5). Transport costs contributed 25.8% of total costs in the overall sample, 26.3% in the diabetes‐only group, and 25.5% in the comorbid group (Tables 4, 5, 6).

**Table 4:** Overall mean and median annual costs of diabetes care at five public facilities in Kenya (2017 US$)

Care‐seeking Episode	Cost Category	n	Mean KES (95% CI)	Median KES (IQR)	Mean US$ (95% CI)	Median US$ (IQR)	As a % of Total Direct Costs
Sick visit	Direct health care costs						
	User charges	59	7435.8 (2611.2‐12 260.4)	4549.2 (1173.0‐6497.4)	72.9 (25.6‐120.2)	44.6 (11.5‐63.7)	23.9
	Medicines	59	15 340.8 (6120‐24 561.6)	5202.0 (1815.6‐12 872.4)	150.4 (60.0‐240.8)	51.0 (17.8‐126.2)	49.3
	Direct non–health care costs						
	Transport	59	8037.6 (4845‐11 220)	5202 (1815.6‐9098.4)	78.8 (47.5‐110.0)	51.0 (17.8‐89.2)	25.8
	Food	59	326.4 (10.2‐632.4)	0	3.2 (0.1‐6.2)	0	1.0
	Subtotal (direct costs)	59	31 130.4 (18 961.8‐43 299.0)	17 156.4 (8578.2‐32 762.4)	305.2 (185.9‐424.5)	168.2 (84.1‐321.2)	
	Indirect costs	59	7466.4 (6262.8‐8659.8)	6344.4 (3906.6‐8884.2)	73.2 (61.4‐84.9)	62.2 (38.3‐87.1)	
	Direct + Indirect costs	59	38 596.8 (25 928.4‐51 265.2)	24 388.2 (12 923.4‐41 524.2)	378.4 (254.2‐502.6)	239.1 (126.7‐407.1)	
In‐patient admission	Direct health care costs						
	User charges	16	16 717.8 (3264.0‐30 161.4)	7170.6 (2203.2‐15 330.6)	163.9 (32.0‐295.7)	70.3 (21.6‐150.3)	64.5
	Medicines	11	4008.6 (1366.8‐6660.6)	3468 (683.4‐8496.6)	39.3 (13.4‐65.3)	34.0 (6.7‐83.3)	10.6
	Direct non–health care costs						
	Transport	36	1917.6 (703.8‐3131.4)	938.4 (418.2‐1734.0)	18.8 (6.9‐30.7)	9.2 (4.1‐17.0)	16.6
	Food	17	2019.6 (204.0‐3825.0)	795.6 (499.8‐1999.2)	19.8 (2.0‐37.5)	7.8 (4.9‐19.6)	8.3
	Subtotal (direct costs)	36	11 526.0 (3825‐19 216.8)	2478.6 (765.0‐14 932.8)	113.0 (37.5‐188.4)	24.3 (7.5‐146.4)	
	Indirect costs	37	4804.2 (3376.2‐6242.4)	3417.0 (1662.6‐6058.8)	47.1 (33.1‐61.2)	33.5 (16.3‐59.4)	
	Direct + Indirect costs	37	16 014.0 (7548.0‐24 490.2)	6507.6 (3498.6‐20 094.0)	157.0 (74.0‐240.1)	63.8 (34.3‐197.0)	
Medicine collection	Direct health care costs						
	Medicines	135	13 300.8 (9608.4‐16 983.0)	5997.6 (3243.6‐13 198.8)	130.4 (94.2‐166.5)	58.8 (31.8‐129.4)	87.3
	Direct non–health care costs						
	Transport	22	9690.0 (5385.6‐13 984.2)	7201.2 (2397.0‐14 402.4)	95.0 (52.8‐137.1)	70.6 (23.5‐141.2)	10.4
	Food	8	5936.4 (4304.4‐7568.4)	7201.2 (4845.0‐7201.2)	58.2 (42.2‐74.2)	70.6 (47.5‐70.6)	2.3
	Subtotal (direct costs)	136	15 116.4 (11 189.4‐19 043.4)	8404.8 (3600.6‐15 595.8)	148.2 (109.7‐186.7)	82.4 (35.3‐152.9)	
	Indirect costs	163	8017.2 (6446.4‐9598.2)	5273.4 (3419.2‐9373.8)	78.6 (63.2‐94.1)	51.7 (33.5‐91.9)	
	Direct + Indirect costs	163	20 634.6 (16 503.6‐24 765.6)	12 739.8 (5803.8‐23 133.6)	202.3 (161.8‐242.8)	124.9 (56.9‐226.8)	
Diagnostic visit	Direct health care costs						
	Test cost	118	5222.4 (2029.8‐8404.8)	1795.2 (1795.2‐3600.6)	51.2 (19.9‐82.4)	17.6 (17.6‐35.3)	80.8
	Direct non–health care costs						
	Transport	21	5844.6 (4192.2‐7486.8)	4804.2 (2397.0‐9598.2)	57.3 (41.1‐73.4)	47.1 (23.5‐94.1)	16.1
	Food	7	3396.6 (1601.4‐5181.6)	3600.6 (2397.0‐3600.6)	33.3 (15.7‐50.8)	35.3 (23.5‐35.3)	3.1
	Subtotal (direct costs)	122	6252.6 (3111.0‐9384.0)	1795.2 (1795.2‐3600.6)	61.3 (30.5‐92.0)	17.6 (17.6‐35.3)	
	Indirect costs	161	4885.8 (4222.8‐5548.8)	3886.2 (2641.8‐5865.0)	47.9 (41.4‐54.4)	38.1 (25.9‐57.5)	
	Direct + Indirect costs	161	9618.6 (7068.6‐12 168.6)	5661.0 (3825.0‐9455.4)	94.3 (69.3‐119.3)	55.5 (37.5‐92.7)	
Scheduled clinics	Direct health care costs						
	User charges	128	4610.4 (2723.4‐6507.6)	2998.8 (2397.0‐4141.2)	45.2 (26.7‐63.8)	29.4 (23.5‐40.6)	24.1
	Medicines	122	10 159.2 (7170.6‐13 147.8)	5967.0 (2397.0‐11 995.2)	99.6 (70.3‐128.9)	58.5 (23.5‐117.6)	50.5
	Direct non–health care costs						
	Transport	144	3957.6 (3325.2‐4579.8)	2397.0 (1203.6‐4804.2)	38.8 (32.6‐44.9)	23.5 (11.8‐47.1)	23.2
	Food	11	4834.8 (397.8‐9271.8)	2397.0 (1795.2‐4804.2)	47.4 (3.9‐90.9)	23.5 (17.6‐47.1)	2.2
	Subtotal (direct costs)	163	15 045.0 (12 056.4‐18 023.4)	9843.0 (4804.2‐17 401.2)	147.5 (118.2‐176.7)	96.5 (47.1‐170.6)	
	Indirect costs	163	4396.2 (3957.6‐4824.6)	3519.0 (2611.2‐5865.0)	43.1 (38.8‐47.3)	34.5 (25.6‐57.5)	
	Direct + Indirect costs	163	19 441.2 (16 207.8‐22 664.4)	14 259.6 (7476.6‐23 878.2)	190.6 (158.9‐222.2)	139.8 (73.3‐234.1)	
Overall costs	Direct health care costs						
	User charges	135	11 413.8 (6987.0‐15 840.6)	6293.4 (5467.2‐9251.4)	111.9 (68.5‐155.3)	61.7 (53.6‐90.7)	17.5
	Medicines	138	33 374.4 (25 092.0‐41 656.8)	19 818.6 (8404.8‐38 403.0)	327.2 (246.0‐408.4)	194.3 (82.4‐376.5)	52.4
	Direct non–health care costs						
	Transport	147	12 168.6 (9894.0‐14 433.0)	7599.0 (3702.6‐14 800.2)	119.3 (97.0‐141.5)	74.5 (36.3‐145.1)	22.6
	Food	31	6232.2 (2743.8‐9720.6)	2397.0 (7956.0‐6497.4)	61.1 (26.9‐95.3)	23.5 (78.0‐63.7)	7.5
	Subtotal (direct costs)	163	53 907.0 (43 625.4‐64 188.6)	35 802.0 (16 462.8‐59 884.2)	528.5 (427.7‐629.3)	351.0 (161.4‐587.1)	
	Indirect costs	163	23 174.4 (20 910.0‐25 438.8)	19 216.8 (13 963.8‐28 417.2)	227.2 (205.0‐249.4)	188.4 (136.9‐278.6)	
	Direct + Indirect costs	163	77 081.4 (65 820.6‐88 342.2)	58 415.4 (33 966.0‐90 922.8)	755.7 (645.3‐866.1)	572.7 (333.0‐891.4)	

**Table 5:** Mean and median annual cost for diabetes‐only patients at five public facilities in Kenya (2017 US$)

Care‐seeking Episode	Cost Category	n	Mean KES (95% CI)	Median KES (IQR)	Mean US$ (95% CI)	Median US$ (IQR)	As a % of Total Direct Costs
Sick visit	Direct health care costs						
	User charges	20	12 733.5 (1825.0‐27 292.0)	3055.0 (845.0‐6110.0)	124.8 (17.9‐267.6)	30.0 (8.3‐59.9)	40.6
	Medicines	20	10 387 (488.6‐20 305.4)	2340 (520‐9425)	101.8 (4.8‐199.1)	22.9 (5.1‐92.4)	33.1
	Direct non–health care costs						
	Transport	20	8268 (3090.8‐13 445.2)	3120 (1560‐9100)	81.1 (30.3‐131.8)	30.6 (15.3‐89.2)	26.3
	Food	20	0	0	0	0	0.0
	Subtotal (direct costs)	20	31 388.5 (7168.3‐55 608.7)	10 335 (6630‐33 605)	307.7 (70.3‐545.2)	101.3 (65.0‐329.5)	
	Indirect costs	20	7360.5 (4925.0‐9796.1)	4545.6 (3860.0‐10 713.2)	72.2 (48.3‐96.0)	44.6 (37.8‐105.0)	
	Direct + Indirect costs	20	38 749.0 (14 080.6‐63 417.5)	18 804.3 (11 197.4‐42 818.0)	379.9 (138.0‐621.7)	184.4 (109.8‐419.8)	
In‐patient admission	Direct health care costs						
	User charges	7	18 568.6 (11 234.9‐48 372.1)	4700 (3150‐16 000)	182.0 (110.1‐474.2)	46.1 (30.9‐156.9)	76.8
	Medicines	5	3372 (1624.9‐8368.9)	1200 (1110‐4350)	33.1 (15.9‐82.0)	11.8 (10.9‐42.6)	10.0
	Direct non–health care costs						
	Transport	15	910.7 (225.3‐1596.1)	600 (200‐1080)	8.9 (2.2‐15.6)	5.9 (2.0‐10.6)	8.1
	Food	6	1441.7 (265.4‐2618.0)	850 (750‐2500)	14.1 (2.6‐25.7)	8.3 (7.4‐24.5)	5.1
	Subtotal (direct costs)	15	11 276.7 (3958.3‐26 511.7)	1860 (400‐7130)	110.6 (38.8‐259.9)	18.2 (3.9‐69.9)	
	Indirect costs	15	5060.3 (2042.4‐8078.2)	3101.0 (1611.6‐6006.7)	49.6 (20.0‐79.2)	30.4 (15.8‐58.9)	
	Direct + Indirect costs	15	16 337.0 (1514.0‐34 187.9)	5968.5 (2567.1‐14 181.8)	160.2 (14.8‐335.2)	58.5 (25.2‐139.0)	
Medicine collection	Direct health care costs						
	Medicines	57	9230.5 (6253.9‐12 207.1)	5040 (2400‐10 680)	90.5 (61.3‐119.7)	49.4 (23.5‐104.7)	84.7
	Direct non–health care costs						
	Transport	8	10 950 (581.6‐22 481.6)	6000 (2400‐13 800)	107.4 (5.7‐220.4)	58.8 (23.5‐135.3)	14.1
	Food	1	7200	7200	70.6	70.6	1.2
	Subtotal (direct costs)	57	10 893.7 (6884.9‐14 902.5)	6000 (3360‐12 000)	106.8 (67.5‐146.1)	58.8 (32.9‐117.6)	
	Indirect costs	69	7771.4 (4800.1‐10 742.7)	4688.2 (3223.1‐7325.3)	76.2 (47.1‐105.3)	46.0 (31.6‐71.8)	
	Direct + Indirect costs	69	16 770.6 (11 672.9‐21 868.2)	10 395.2 (5225.2‐16 739.7)	164.4 (114.4‐214.4)	101.9 (51.2‐164.1)	
Diagnostic visit	Direct health care costs						
	Test cost	47	7598.3 (201.4‐15 398.0)	1800 (1800‐3600)	74.5 (2.0‐151.0)	17.6 (17.6‐35.3)	90.6
	Direct non–health care costs						
	Transport	7	4971.4 (2075.4‐7867.5)	4800 (2400‐7200)	48.7 (20.3‐77.1)	47.1 (23.5‐70.6)	8.8
	Food	1	2400	2400	23.5	23.5	0.6
	Subtotal (direct costs)	50	7886.4 (572.7‐15 200.1)	1800 (1800‐3600)	77.3 (5.6‐149.0)	17.6 (17.6‐35.3)	
	Indirect costs	68	4423.4 (3644.7‐5202.1)	3850.2 (2607.8‐4887.4)	43.4 (35.7‐51.0)	37.7 (25.6‐47.9)	
	Direct + Indirect costs	68	10 222.2 (4827.4‐15 616.9)	5682.4 (3809.1‐9273.8)	100.2 (47.3‐153.1)	55.7 (37.3‐90.9)	
Scheduled clinics	Direct health care costs						
	User charges	55	6362.2 (1953.2‐10 771.1)	3000 (2400‐4080)	62.4 (19.1‐105.6)	29.4 (23.5‐40.0)	38.6
	Medicines	50	6289.2 (4546.0‐8032.4)	4350 (2400‐9000)	61.7 (44.6‐78.7)	42.6 (23.5‐88.2)	34.7
	Direct non–health care costs	69	13 148.7 (9166.8‐17 130.6)	9000 (4080‐17 640)	128.9 (89.9‐167.9)	88.2 (40‐172.9)	
	Transport	62	3878.7 (2987.6‐4769.8)	2400 (1200‐4800)	38.0 (29.3‐46.8)	23.5 (11.8‐47.1)	26.5
	Food	1	2400	2400	23.5	23.5	0.3
	Subtotal (direct costs)	69	13 148.7 (9166.8‐17 130.6)	9000 (4080‐17 640)	128.9 (89.9‐167.9)	88.2 (40‐172.9)	
	Indirect costs	69	4071.1 (3542.5‐4599.6)	3516.1 (2344.1‐5086.7)	39.9 (34.7‐45.1)	34.5 (23.0‐49.9)	
	Direct + Indirect costs	69	17 219.8 (13 072.6‐21 366.9)	12 792.3 (6876.1‐21 156.1)	168.8 (128.2‐209.5)	125.4 (67.4‐207.4)	
Overall costs	Direct health care costs						
	User charges	58	14 839.1 (4730.7‐24 947.6)	6250 (5000‐7880)	145.5 (46.4‐244.6)	61.3 (49.0‐77.3)	30.3
	Medicines	57	22 275.3 (16 647.7‐27 902.8)	18 400 (7400‐30 500)	218.4 (163.2‐273.6)	180.4 (75.5‐299.0)	44.0
	Direct non–health care costs						
	Transport	63	11 218.4 (7919.4‐14 517.5)	6300 (3360‐14 640)	110.0 (77.6‐142.3)	61.8 (32.9‐143.5)	24.7
	Food	9	3016.7 (609.4‐5423.9)	2400 (800‐3200)	29.6 (6.0‐53.2)	23.5 (7.8‐31.4)	1.0
	Subtotal (direct costs)	69	46 754.4 (31 008.3‐62 500.4)	31 500 (13 880‐50 840)	458.4 (304.0‐612.7)	308.8 (136.1‐498.4)	
	Indirect costs	64	21 861.1 (18 070.1‐25 652.1)	17 483.0 (13 136.7‐26 187.8)	214.3 (177.2‐251.5)	171.4 (128.8‐256.7)	
	Direct + Indirect costs	69	68 615.5 (51472.6‐85 758.3)	52 372.5 (28 458.9‐75 288.0)	672.7 (504.6‐840.8)	513.5 (279.0‐738.1)	

**Table 6:** Mean and median annual cost for comorbid (diabetes mellitus and hypertension) patients at five public facilities in Kenya (2017 US$)

Care‐seeking Episode	Cost Category	n	Mean KES (95% CI)	Median KES (IQR)	Mean US$ (95% CI)	Median US$ (IQR)	As a % of Total Direct Costs
Sick visit	Direct health care costs						
	User charges	39	4720 (3405.9‐6034.1)	5200 (1300‐6500)	46.3 (33.4‐59.2)	51.0 (12.7‐63.7)	15.2
	Medicines	39	17 880 (4641.7‐31 118.3)	7670 (3120‐15 600)	175.3 (45.5‐305.1)	75.2 (30.6‐152.9)	57.7
	Direct non–health care costs						
	Transport	39	7913.3 (3726.5‐12 100.2)	5200 (2600‐9100)	77.6 (36.5‐118.6)	51.0 (25.5‐89.2)	25.5
	Food	39	486.7 (22.5‐950.8)	0	4.8 (0.2‐9.3)	0	1.6
	Subtotal (direct costs)	39	31 000 (16 546.6‐45 453.4)	20 800 (10 660‐32 760)	303.9 (162.2‐445.6)	203.9 (104.5‐321.2)	
	Indirect costs	39	7515.4 (6110.2‐8920.6)	6856.5 (4063.1‐8888.0)	73.7 (59.9‐87.5)	67.2 (39.8‐87.1)	
	Direct + Indirect costs	39	38 515.4 (23 247.1‐53 783.7)	29 688.0 (15 563.4‐41 037.2)	377.6 (227.9‐527.3)	291.1 (152.6‐402.3)	
In‐patient admission	Direct health care costs						
	User charges	9	15 273.9 (287.2‐30 834.9)	9990 (1000‐14 660)	149.7 (2.8‐302.3)	97.9 (9.8‐143.7)	56.0
	Medicines	6	4545.8 (166.7‐8924.9)	3598.5 (680‐8500)	44.6 (1.6‐87.5)	35.3 (6.7‐83.3)	11.1
	Direct non–health care costs						
	Transport	21	2633.3 (592.3‐4674.4)	1200 (800‐2400)	25.8 (5.8‐45.8)	11.8 (7.8‐23.5)	22.5
	Food	11	2331.8 (589.9‐5253.5)	600 (300‐2000)	22.9 (5.8‐51.5)	5.9 (2.9‐19.6)	10.4
	Subtotal (direct costs)	21	11 699.5 (2885.6‐20 513.4)	2750 (1180‐15 960)	114.7 (28.3‐201.1)	27.0 (11.6‐156.5)	
	Indirect costs	22	4633.5 (3108.5‐6158.5)	4248.7 (1660.4‐6187.4)	45.4 (30.5‐60.4)	41.7 (16.3‐60.7)	
	Direct + Indirect costs	22	15 801.2 (6790.6‐24 811.9)	7149.1 (4060.4‐20 634.9)	154.9 (66.6‐243.3)	70.1 (39.8‐202.3)	
Medicine collection	Direct health care costs						
	Medicines	78	16 269.2 (10 291.7‐22 246.8)	7200 (3600‐15 000)	159.5 (100.9‐218.1)	70.6 (35.3‐147.1)	88.4
	Direct non–health care costs						
	Transport	14	8965.7 (4969.6‐12 961.8)	7200 (4800‐14 400)	87.9 (48.7‐127.1)	70.6 (47.1‐141.2)	8.7
	Food	7	5760 (3876.6‐7643.4)	7200 (4800‐7200)	56.5 (38.0‐74.9)	70.6 (47.1 (70.6)	2.8
	Subtotal (direct costs)	79	18 162.5 (12 069.3‐24 255.8)	9120 (4320‐18 000)	178.1 (118.3‐237.8)	89.4 (42.4‐176.5)	
	Indirect costs	94	8205.7 (6515.2‐9896.2)	5860.2 (3809.1‐9962.4)	80.4 (63.9‐97.0)	57.5 (37.3‐97.7)	
	Direct + Indirect costs	94	23 470.0 (17 351.3‐29 588.6)	14 522.6 (6022.2‐25 785.0)	230.1 (170.1‐290.1)	142.4 (59.0‐252.8)	
Diagnostic visit	Direct health care costs						
	Test cost	71	3643.9 (2132.2‐5155.7)	1800 (1800‐3600)	35.7 (20.9‐50.5)	17.6 (17.6‐35.3)	70.3
	Direct non–health care costs						
	Transport	14	6274.3 (4030.7‐8517.8)	7200 (2400‐9600)	61.5 (39.5‐83.5)	70.6 (23.5‐94.1)	23.9
	Food	6	3560 (1390.9‐5729.1)	3600 (2400‐3600)	34.9 (13.6‐56.2)	35.3 (23.5‐35.3)	5.8
	Subtotal (direct costs)	72	5110.0 (3282.5‐6937.4)	1800 (2400‐4080)	50.1 (32.2‐68.0)	17.6 (23.5‐40.0)	
	Indirect costs	93	5220.8 (4221.9‐6219.6)	4102.2 (2695.7‐6153.2)	51.2 (41.4‐61.0)	40.2 (26.4‐60.3)	
	Direct + Indirect costs	93	9176.9 (7078.3‐11 275.4)	5609.1 (4102.2‐10 060.2)	90.0 (69.4‐110.5)	55.0 (40.2‐98.6)	
Scheduled clinics	Direct health care costs						
	User charges	73	3292.6 (2909.4‐3675.7)	3000 (2640‐4200)	32.3 (28.5‐36.0)	29.4 (25.9‐41.2)	15.6
	Medicines	72	12 839.2 (7966.7‐17 711.6)	7200 (3300‐13 200)	125.9 (78.1‐173.6)	70.6 (32.4‐129.4)	59.8
	Direct non–health care costs	94	16 432.2 (12 120.0‐20 744.5)	10 560 (5640‐17 400)	161.1 (118.8‐203.4)	103.5 (55.3‐170.6)	
	Transport	82	4013.3 (3125.5‐4901.0)	2400 (1200‐4800)	39.3 (30.6‐48.0)	23.5 (11.8‐47.1)	21.3
	Food	10	5076 (134.9‐10 017.1)	2640 (1800‐4800)	49.8 (1.3‐98.2)	25.9 (17.6 (47.1)	3.3
	Subtotal (direct costs)	94	16 432.2 (12 120‐20 744.5)	10 560 (5640‐17 400)	161.1 (118.8‐203.4)	103.5 (55.3‐170.6)	
	Indirect costs	94	4632.5 (3985.5‐5279.4)	3516.1 (2695.7‐5860.2)	45.4 (39.1‐51.8)	34.5 (26.4‐57.4)	
	Direct + Indirect costs	94	21 064.7 (16 336.1‐25 793.2)	15 255.1 (8769.4‐24 164.3)	206.5 (160.2‐252.9)	149.6 (86.0‐236.9)	
Overall costs	Direct health care costs						
	User charges	77	8837.2 (6958.2‐10 716.1)	7500 (5500‐9400)	86.6 (68.2‐105.1)	73.5 (53.9‐92.2)	12.9
	Medicines	81	41 187.2 (27 764.2‐54 610.1)	21 900 (10 300‐42 700)	403.8 (272.2‐535.4)	214.7 (101.0‐418.6)	63.3
	Direct non–health care costs						
	Transport	84	12 873.2 (9710.5‐16 035.9)	8340 (3800‐15 300)	126.2 (95.2‐157.2)	81.8 (37.3‐150)	20.5
	Food	24	7438.3 (2701.2‐12 175.4)	2300 (725‐10 100)	72.9 (26.5‐119.4)	22.5 (7.1‐99.0)	3.3
	Subtotal (direct costs)	94	59 161.8 (45 440.9‐72 882.8)	42 750 (17 162‐65 550)	580.0 (445.5‐714.5)	419.1 (168.3‐642.6)	
	Indirect costs	94	24 137.9 (21 314.9‐26 960.9)	20 571.8 (15 090.0‐29 984.8)	236.6 (209.0‐264.3)	201.2 (147.9‐294.0)	
	Direct + Indirect costs	94	83 299.8 (68 223.5‐98 376.0)	63 970.6 (37 833.6‐100 271.8)	816.7 (668.9‐964.5)	627.2 (370.9‐983.1)	

#### 3.2.2 Inpatient costs

Twenty‐two percent (n = 37) of the patients in the study reported an inpatient admission in the past year with each admission lasting a mean of 1.6 days (95% CI, 1.3‐1.9). User charges constituted the highest direct costs attracting an estimated KES 16 718.8 (95% CI, 3264‐30 161.4) (US$ 163.9 [95% CI, 32‐295.7]), KES 18 568.6 (95% CI, 11 234.9‐48 372.1) (US$ 182.0 [95% CI, 110.1‐474.2]), and KES 15 273.9 (95% CI, 287.2‐30 834.9) (US$ 149.7 [95% CI, 2.8‐302.3]) mean annual costs in the overall sample, diabetes‐only patients, and comorbid patients, respectively. Additionally, direct costs were higher than indirect costs in all the three groups (Tables 4, 5, 6).

#### 3.2.3 Medicine collection costs

**Figure 1:**
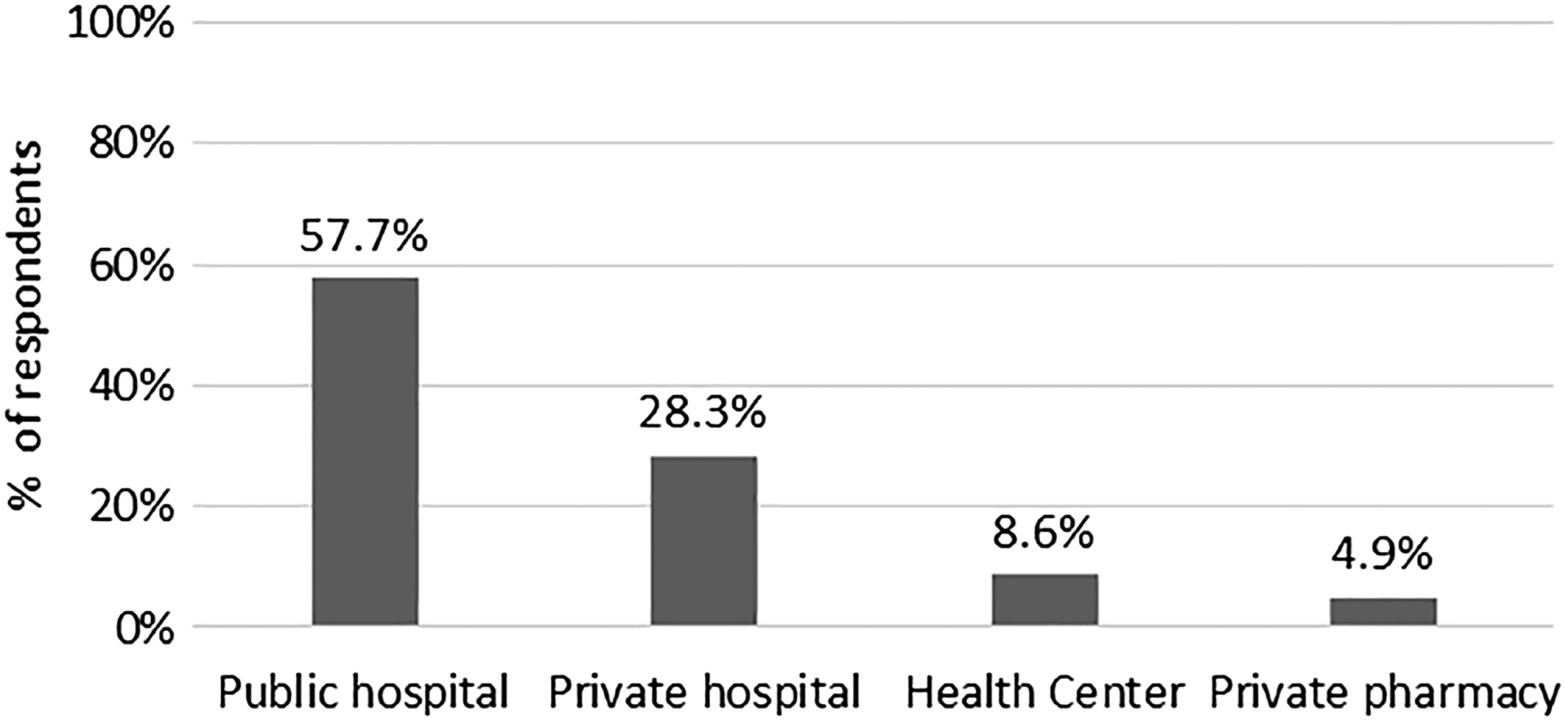
Source of medicines

The median number of routine medicines prescribed to diabetes‐only patients was 2 (IQR 1‐2) and 3 (IQR, 3‐4) for comorbid patients. The most expensive antidiabetic regimen was metformin and insulin (mean annual cost KES 15 636.6 (95% CI, 2723.4‐28 539.6) (US$ 153.3 [95% CI, 26.7‐279.8]) while metformin, glibenclamide, and enalapril combinations were the most expensive medicines prescribed to comorbid patients—mean annual cost KES 18 635.4 (95% CI, 3131.4‐40 402.2) (US$ 182.7 [95% CI, 30.7‐396.1]) (Table 7). In addition, more than half (57.7%) of sampled patients reported obtaining their routine medicines from a public hospital (Figure 1). Medicines accounted for 87.3% of total OOP costs during medicine collection visits while transport and food accounted for 10.4% and 2.3% in the overall sample, respectively (Table 4).

**Table 7:** Medicines costs

Drug Name	n	Mean KES (95% CI)	Median KES (IQR)	Mean US$ (95% CI)	Median US$ (IQR)
Metformin	16	8384.4 (2509.2‐14 259.6)	3172.2 (897.6‐13 198.8)	82.2 (24.6‐139.8)	31.1 (8.8‐129.4)
Metformin + insulin	15	15 636.6 (2723.4‐28 539.6)	10 404.0 (4804.2‐15 004.2)	153.3 (26.7‐279.8)	102.0 (47.1‐147.1)
Metformin + glibenclamide	63	12 678.6 (6201.6‐19 155.6)	3835.2 (1438.2‐12 484.8)	124.3 (60.8‐187.8)	37.6 (14.1‐122.4)
Metformin + glibenclamide + nifedipine	18	17 554.2 (336.6‐34 771.8)	8160.0 (0.0‐14 881.8)	172.0 (3.3‐340.9)	80.0 (0.0‐145.9)
Metformin + glibenclamide + enalapril	15	18 635.4 (3131.4‐40 402.2)	3600.6 (0.0‐16 320.0)	182.7 (30.7‐396.1)	35.3 (0.0‐160.0)
Metformin + glibenclamide + hydrochlorothiazide	21	13 935.6 (1122‐26 270)	5395.8 (1438.2‐13 198.8)	136.6 (11.0‐257.5)	52.9 (14.1‐90.0)
Metformin + nifedipine + hydrochlorothiazide	11	16 218.0 (1530.0‐30 895.8)	5457.0 (1438.2‐13 198.8)	159.0 (15.0‐302.9)	53.5 (14.1‐129.4)
Insulin + other antihypertensives^a^	3	9557.4 (1315.8‐20 430.6)	8404.8 (5875.2‐14 402.4)	93.7 (12.9‐200.3)	82.4 (57.6‐141.2)

a Atenolol + losartan + amlodipine.

#### 3.2.4 Diagnostic/laboratory test costs

The main routine diagnostic tests reported by diabetes‐only patients were fasting blood sugar (FBS) and weight—mean annual cost KES 1499.4 (95% CI, 1030.2‐4029) (US$ 14.7 [95% CI, 10.1‐39.5]) while comorbid patients reported FBS and blood pressure, mean annual cost KES 4518.6 (95% CI, 173.4‐8853.6) (US$ 44.3 [95% CI, 1.7‐86.8]), and echocardiogram and kidney function tests, mean annual cost KES 3335.4 (95% CI, 1081.2‐5589.6) (US$ 32.7 [95% CI, 10.6‐54.8]). The mean annual direct costs for diagnostic/laboratory tests were generally higher for diabetes‐only patients (Table 5) compared with comorbid patients (Table 6). Moreover, 18% (n = 29) of the patients reported incurring costs to purchase of either glucometers or blood pressure monitoring machines, incurring a mean cost of KES 3542.9 (95% CI, 2724.1‐4361.6) (US$ 34.7 [95% CI, 26.7‐42.8]) with additional mean annual cost of KES 16 885.7 (95% CI, 12 377.3‐21 394.1) (US$ 165.5 [95% CI, 121.3‐209.7]) on consumables like glucose strips and lancets.

#### 3.2.5 Scheduled clinic appointment costs

Majority of patients (76.7%, n = 125) attended scheduled clinic visits monthly. Medicines attracted the highest cost in the overall sample, mean annual cost KES 10 159.2 (95% CI, 7170.6‐13 147.8) (US$ 99.6 [95% CI, 70.3‐128.9]), and in the comorbid patients, mean annual cost KES 12 839.2 (95% CI, 7966.7‐17 711.6) (US$ 125.9 [95% CI, 78.1‐173.6]). However, user charges attracted the highest cost, mean annual cost KES 6362.2 (95% CI, 1953.2‐10 771.1) (US$ 62.4 [95% CI, 19.1‐105.6]) in the diabetes‐only group during scheduled clinic appointments. In addition, transport costs accounted for 23.2%, 26.5%, and 21.3% of total direct OOP costs in the overall sample, diabetes‐only, and comorbid patients, respectively (Tables 4, 5, 6).

#### 3.2.6 Overall care‐seeking cost

When costs from all care‐seeking episodes were combined, the average direct annual costs—KES 53 907 (95% CI, 43 625.4‐64 188.6) (US$ 528.5 [95% CI, 427.7‐629.3])—was higher than the average indirect annual costs—KES 23 174.4 (95% CI, 20 910.0‐25 438.8) (US$ 227.2 [95% CI, 205.0‐249.4])—in the overall sample. Of note, medicine costs in the overall sample, in the diabetes‐only patients, and in the comorbid patients attracted the highest costs. This was closely followed by transport costs in the overall sample and in the comorbid patients while user charges were the second highest cost category after medicines in the diabetes‐only group (Tables 4, 5, 6).

### 3.3 Impact on household income and coping strategies

**Figure 2:**
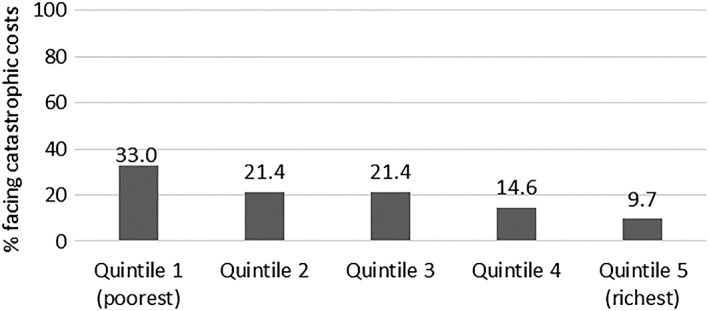
Relationship between catastrophic costs and socio‐economic status

**Figure 3:**
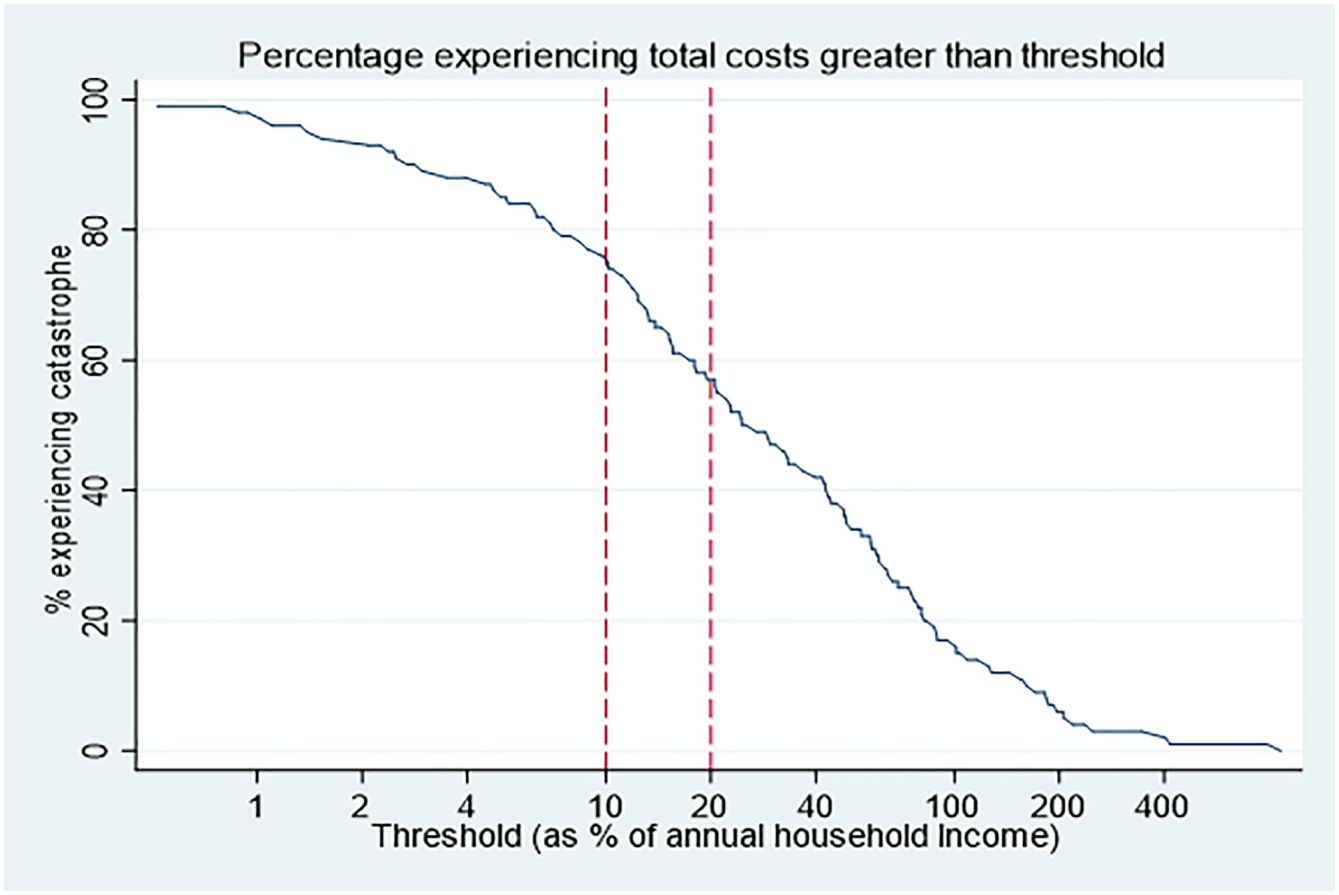
Percentage experiencing total costs greater than threshold [Colour figure can be viewed at http://wileyonlinelibrary.com]

Costs for DM care services was catastrophic to three quarters (n = 123), 75.5% (95% CI, 68.3‐82.1) of patients at the 10% annual household income threshold. Moreover, comorbid patients (n = 94) realized higher catastrophic costs, 79.8% (95% CI, 71.5‐88.1), compared with diabetes‐only patients (n = 69), 69.6% (95% CI, 58.4‐80.7). Alternatively, when transport costs were excluded, (n = 103) 63.2% (95% CI, 55.7‐70.7) of patients incurred catastrophic costs. Among patients experiencing catastrophe, patients in the lowest wealth quintile incurred higher direct costs with few resources to meet the health care costs (Figure 2). Nonetheless, Figure 3 shows a decreasing proportion of patients experiencing catastrophic costs if the 10% annual income threshold was increased. Patients had to borrow (23.3%) from friends/family, sell an asset (29.9%), and use savings (36.8%) to pay for DM care costs.

### 3.4 Productivity and social impact of diabetes mellitus

Patients were asked to report any work days missed due to DM illness in the last 3 months before the survey. Of the 163 patients, 33% reported to miss a median of 21 (IQR, 7‐60) working days. Forty‐five percent of the respondents reported disrupted social life due to DM, affecting sexual life (n = 11), job loss (n = 48), divorce (n = 4), and separation from spouse (n = 6).

## 4 DISCUSSION

This study has estimated patient costs for adults with DM that sought care at five public health care facilities in two counties in Kenya. The study's main finding is that patient cost for DM are driven by medicine expenses. Similar findings have been reported by recent studies in Kenya and South Africa.^19^, ^34^ Costs due to medicine have been shown to reduce adherence to medication and demand for health services by patients with NCDs.^35^ Past studies conducted in LMICs have however shown that social health insurance schemes do not comprehensively cover the costs for medicines^36^ and that OOP costs, which are majorly contributed by medicines, are a hindrance to attainment of universal health coverage in many low resource settings.^37^, ^38^ Indeed, any reductions or removal of medicine costs is likely to increase access to DM health care services, but additional resources will be required to cover any concomitant increase in service utilization.

The incidence of catastrophic costs documented in this study is arguably high and suggests that DM care in the sampled health facilities is unaffordable to majority of patients, especially those in the lowest wealth quintile whose capacity to pay is limited compared with those in higher socio‐economic group. This is a concern given the high poverty rates in Kenya (36.1%) and that only 19% of Kenya's population have a form of health insurance.^39^, ^40^ Furthermore, a past study has shown that families with a member with an NCD incurs three times higher costs compared with families without a member with an NCD.^17^ Our study has also shown that DM patients reporting hypertension comorbidity incur higher costs overall compared with diabetes‐only patients. This places additional financial burden on families of these patients, similar to findings of previous studies.^18^, ^41^

Our results indicate that transport cost offers an access barrier to DM patients given that it takes a significant proportion of total direct costs in all care‐seeking episodes. In part, the high transport costs reported in this study can be attributed to poor quality of care in public health care facilities. For instance, 48.9% of the patients reported lack of medicines and diagnostic facilities as a reason for not visiting nearest facilities. This phenomenon has been observed from studies in Uganda and Zambia.^42^, ^43^ Prior studies conducted in Kenya highlight that transport costs are a key access barrier especially to poor patients.^38^, ^44^ Moreover, 36% of patients in the overall sample reported a sick visit outside of scheduled clinic appointments incurring an annual mean cost of KES 38 597 (US$ 378.4). Failure in health‐care delivery has been shown to increase the risk of catastrophe, exacerbate socio‐economic iniquities, and reduce the probability of comprehensive treatment.^45^ These findings therefore reiterate the need for policymakers to develop mechanisms to improve quality of care for diabetic patients in public health facilities since this has serious cost implications on patients. Additionally, since more than three quarters (76.7%) of the patients reported attending their routine scheduled clinics monthly, introducing mechanisms to minimize facility visits, for example, by enhancing and supporting self‐care by patients is likely to reduce transport costs.

The indirect costs due DM care in this study are noteworthy. For instance, of the 163 patients enrolled in the study, 48 (29.5%) had to stop working because of DM and 54 (33%) reported a median loss of 21 working days over the past 90‐day period, which is equal to 1 month's wage in the informal sector in Kenya. Similarly, the overall mean indirect costs in all care‐seeking episodes was KES 23 174 (US$ 227.2) and were primarily contributed by long waiting times at health facilities. It has been shown that long‐waiting times while receiving care reduces demand for chronic care services.^46^ To achieve optimal efficiency and increase demand for service delivery for patients with chronic illnesses like diabetes, there is urgent need to redesign health service delivery for these patients with a view to making care more patient‐centred to meet the unique needs of these patients.

## 5 LIMITATIONS

Our study had some limitations. Due to the recall periods, results may be subject to recall bias. Our study only focused on those who utilized health services, thus excluding those with undiagnosed diabetes who may still incur costs due to symptoms associated with diabetes. Also, costs reported in our study could be potentially overestimated since patients were selected purposefully. Consequently, our findings are not nationally representative. Also, our study relied on the diagnosis reported by patients, hence could not distinguish between type 1 and type 2 diabetes. Use of an official minimum wage to estimate productivity losses for all patients could have potentially overestimated indirect costs among patients who were unemployed prior to their illness or underestimated indirect costs among those who were employed.^30^ These limitations notwithstanding, the findings presented are potentially useful as inputs in costing and/or cost‐effectiveness models that require patient cost and suggest there are significant OOP costs associated with DM management in public facilities in Kenya, which offer a barrier of access to care.

## AUTHOR CONTRIBUTIONS

Edwine Barasa, Anthony Etyang, Kenneth Munge, Jane Mbui, Zipporah Bukania, Fredrick Kirui, and Andrew Obala conceived the study. Robinson Oyando, Martin Njoroge, Antipa Sigilai, Kenneth Munge, Peter Nguhiu, and Edwine Barasa contributed to the development of data collection tools. Robinson Oyando collected the data. Robinson Oyando and Edwine Barasa analysed the data. Robinson Oyando developed the first draft of the manuscript. All authors contributed to writing subsequent versions of the manuscript.

## CONFLICT OF INTEREST

The authors declare that they have no competing interests.

## FUNDING SOURCE

This work is funded by a KEMRI internal research grant (IRG‐04) awarded to Anthony Etyang. Additional funds from a Wellcome Trust research training fellowship (#107527) awarded to Edwine Barasa, a Wellcome Trust International Master's Fellowship (#214622) awarded to Robinson Oyando, and a Wellcome Trust core grant (#092654) awarded to the KEMRI‐Wellcome Trust research program supported this work.
